# SFXN1 Reduction Alleviates Cerebral Ischemia–Reperfusion Injury by Promoting Neuronal Survival and Reducing Neuroinflammation

**DOI:** 10.1111/cns.70457

**Published:** 2025-05-26

**Authors:** Xiangyu Xu, Zhongying Duan, Xin Zhou, Rui Zhao, Jing Xu, Zhaolong Zhang, Mengfei Lv, Qi Wan, Yu Cui

**Affiliations:** ^1^ Institute of Neuroregeneration and Neurorehabilitation Qingdao Medical College, Qingdao University Qingdao Shandong China; ^2^ School of Basic Medicine Qingdao Medical College, Qingdao University Qingdao Shandong China; ^3^ Department of Interventional Radiology The Affiliated Hospital of Qingdao University Qingdao Shandong China; ^4^ Shandong First Medical University & Shandong Academy of Medical Sciences Jinan Shandong China; ^5^ Faculty of Life and Health Shenzhen University of Advanced Science Shenzhen China

**Keywords:** ischemic–reperfusion injury, microglia, mitochondria, neuroinflammation, neurons, ROS, serine, SFXN1

## Abstract

**Aim:**

Sideroflexin 1 (SFXN1) is an important inner mitochondrial membrane protein that regulates many physiological and pathological events. However, the role of SFXN1 in cerebral ischemia–reperfusion (I/R)‐induced neuronal death remains unclear.

**Methods:**

We employed in vivo injury models of transient middle cerebral artery occlusion (tMCAO) and in vitro models of lipopolysaccharide (LPS) stimulation and oxygen–glucose deprivation/reperfusion (OGD/R) to investigate the regulatory effects of SFXN1 on neuroinflammation and brain injury. Western blotting, immunofluorescence, and real‐time quantitative PCR were utilized to assess SFXN1 expression, proinflammatory signaling pathways activation, and cytokine levels in vitro. Cerebral infarction was evaluated using 2,3,5‐triphenyltetrazolium chloride (TTC) staining and Nissl staining.

**Results:**

SFXN1 expression was upregulated following cerebral I/R injury. Both neurons and microglia exhibited increased SFXN1 expression after oxygen–glucose deprivation/reoxygenation (OGD/R) treatment. SFXN1 knockdown reduced OGD/R‐induced neuronal death and alleviated cerebral I/R injury. Additionally, conditioned medium from SFXN1‐knockdown microglia reduced neurotoxicity in vitro. Mechanistically, SFXN1 induced mitochondrial dysfunction and neuronal death after OGD/R in an iron‐independent manner. Furthermore, SFXN1 promoted the production of proinflammatory cytokines by promoting NF‐κB activation, partially through iron transport in microglia after OGD/R.

**Conclusion:**

This study reveals the novel role of SFXN1 in exacerbating cerebral I/R injury by reducing neuronal survival through the modulation of mitochondrial function and promotion of microglia‐mediated neuroinflammation via NF‐κB activation.

## Graphical abstract

After cerebral ischemia/reperfusion (I/R), the expression of SFXN1 is upregulated in both neurons and microglia, exacerbating brain infarction. In neurons, increased SFXN1 expression induces mitochondrial dysfunction in an iron‐independent manner. In microglia, SFXN1 promotes the production of pro‐inflammatory cytokines by enhancing NF‐κB activation, partially through iron transport.

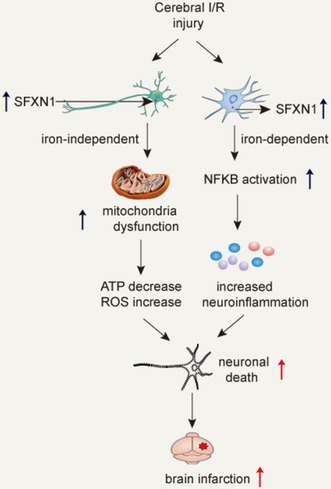

## 1 Introduction

Ischemic stroke is one of the most devastating neurological diseases and often leads to severe and permanent disability. Currently, intra‐arterial therapy and tissue plasminogen activator thrombolysis are the only effective treatments for ischemic stroke [1, 2, 3]. However, ischemia/reperfusion‐induced injury occurs during these therapies, which may further exacerbate neuronal death [4, 5]. Therefore, elucidating the molecular mechanism of ischemia/reperfusion‐induced brain injury is essential for improving therapeutic strategies and developing new drugs for treating ischemic stroke.

Various mechanisms contributing to I/R‐induced neuronal death, including neuronal apoptosis, necrosis, excitotoxicity, and oxidative stress, have been identified [6, 7]. Mitochondria, the powerhouse of the cell, play a critical role in neuronal energy homeostasis and are inevitably involved in neuronal death caused by ischemia [8, 9]. Oxygen and glucose deprivation can induce mitochondrial dysfunction within minutes after ischemia, leading to limited ATP production and the accumulation of reactive oxidative species (ROS) [10]. Previous studies have explored the molecular regulation of mitochondrial events, such as ATP production, ROS production, mitochondrial membrane potential depolarization, and mitochondrial fission and fusion, following ischemia stroke [11]. However, the role of mitochondrial carriers in ischemic stroke remains poorly understood.

Postischemic inflammation occurs in all stages of cerebral I/R injury [6, 12]. As the resident macrophages of the central nervous system, microglia respond to damage‐associated molecular patterns (DAMPs) such as HMGB1, ATP, and HSPs released from damaged brain tissue. This response initiates neuroinflammation which influences brain injury and regeneration [13, 14, 15]. Many metabolism‐related enzymes and regulators have been reported to modulate the inflammatory response of microglia [16, 17, 18, 19], but the role of mitochondrial carriers in microglia‐mediated neuroinflammation remains largely unknown.

Sideroflexin 1 (SFXN1) is a highly conserved multispanning transmembrane protein localized in the inner mitochondrial membrane [11]. It has been implicated in anemia, erythrocyte maturation, and tumorigenesis [11, 20, 21, 22]. Molecular studies suggest that SFXN1 functions as a transporter of mitochondrial metabolites, including serine and iron, to regulate mitochondrial metabolism, iron homeostasis, and heme biosynthesis [23, 24, 25]. Loss of SFXN1 compromises mitochondrial complex III biogenesis, impairs respiratory chain activity, and reduces coenzyme Q levels [24]. Although previous studies have linked decreased SFXN1 expression in the brain to Alzheimer's disease and Parkinson's disease, and SFXN1 downregulation has been associated with nonviral hepatocellular carcinoma [26, 27, 28], the role of SFXN1 in neuronal death remains unclear.

In this study, we found that the expression of SFXN1 was upregulated in neurons and microglia following OGD/R. SFXN1 knockdown reduced neuronal death after OGD/R by improving mitochondrial function in an iron‐independent manner. In addition, SFXN1 knockdown attenuated neuroinflammation in microglia after OGD/R by suppressing the activation of the NF‐κB signaling pathway in an iron‐dependent manner. Virus‐mediated knockdown of SFXN1 in vivo reduced brain infarction after ischemic stroke.

## 2 Materials and Methods

### 2.1 Mice and tMCAO Model

Adult male C57BL/6 mice weighing 25–30 g were purchased from Jinan Pengyue Experimental Animal Breeding Co. Ltd. for transient middle cerebral artery occlusion (tMCAO). As previous studies showed that female rodents exhibit less brain damage compared with their male counterparts in MACO due to the existence of endogenous ovarian steroids [29, 30], most of the current study prefers to use male mice to investigate the effect on I/R‐induced injury [31, 32, 33]. All animal experiments were carried out following the guidelines of the National Institutes of Health and were approved by the institutional animal care and use committee of Qingdao University.

Transient focal cerebral ischemia was induced in adult mice via suture occlusion techniques [33, 34]. In brief, C57BL/6 mice were anesthetized with 1.5% isoflurane in a 70% N_2_O/30% O_2_ mixture using an anesthesia mask. To occlude the left middle cerebral artery, a nylon suture was inserted from the external carotid artery into the left internal carotid artery. After occlusion for 90 min, the filament was removed for reperfusion, and the left external carotid arteries were ligated to recover the wound. The same surgery as that in the MCAO group was performed in the sham group, except for the immediate insertion and removal of monofilaments. Laser Doppler flowmetry (PERIMED PSI‐Z) was used to detect the brain blood flow changes. Animals were excluded from further experiments if no significant reduction in regional cerebral blood flow was detected after ischemia. During the operation, mice were resuscitated on top of a warming pad (RWD, 69003). The respiration of the mice was monitored using a breathing machine. The mice were placed back in the heated cage during the recovery phase with ad libitum access to food and water. After cerebral ischemia–reperfusion injury in mice, the penumbra is located in the cortical region surrounding the ischemic core. On the basis of this anatomical localization, the cortical tissue adjacent to the infarct core is extracted for subsequent detection.

### 2.2 Cell Culture

The SH‐SY5Y and BV2 cell lines were cultured in DMEM (Solarbio, China) containing 10% fetal bovine serum (Gibco, USA) and 1% penicillin/streptomycin (Gibco, USA).

Cortical neurons [35, 36], primary microglia, and primary astrocyte [37, 38] culture protocols were adapted from previous literature (Supporting Information).

### 2.3 TTC Staining

The 2,3,5‐triphenyltetrazoliumchloride staining (TTC) method was used to measure the infarct volume of the mice brain as described previously [39, 40]. At 24 h after tMCAO, the mice were perfused with ice‐cold 0.9% saline, and the brains were harvested rapidly. The brains were then cooled, cut into coronal segments, and stained with 2% TTC in phosphate‐buffered saline at 37°C in the dark for 30 min. Finally, the slices were fixed for at least 24 h in 4% paraformaldehyde at 4°C and then scanned. The infarct volume was analyzed using Image J analysis software [41]. Percent of infarction was calculated as previously described to compensate for edema formation [40, 42]. The normal volume of the contralateral hemisphere and the normal volume of the ipsilateral hemisphere were measured, and the infarct percentage was calculated as the percentage of contralateral structure to correct the possible interference of cerebral edema [39].

### 2.4 Oxygen–Glucose Deprivation/Reoxygenation (OGD/R)

To establish OGD conditions, cells were transferred to a glucose‐free extracellular solution (116 mM NaCl, 0.8 mM MgSO_4_, 5.4 mM KCl, 1.0 mM NaH_2_PO_4_, 26 mM NaHCO_3_, and 1.8 mM CaCl_2_) and placed into the anaerobic incubator (CONCEPT400, RUSKIN, UK) and held in 95% N_2_/5% H_2_ at 37°C for indicated times. After the medium was replaced with normal culturing medium, the cells were reoxygenated in a 5% CO_2_/95% O_2_ incubator for the indicated time [39]. The OGD time for primary neurons, microglia, and astrocytes is 1.5 h. The OGD time for the SH‐SY5Y cell line is 4 h, and for BV2 microglial cells is 2 h.

### 2.5 siRNA‐Mediated Interference

Lipofectamine RNAiMAX Transfection Reagent (Thermo Fisher Scientific, USA) was mixed with gene‐specific targeting siRNA (20 nM) and then transfected into the indicated cells according to the manufacturer's protocol [38]. After 48 h, cells were used for OGD/R treatment or LPS stimulation and then harvested for further experiments.

### 2.6 Cell Viability Assay

A Cell Counting Kit‐8 (Solarbio, China) was used to evaluate cell viability. Briefly, neurons or SH‐SY5Y cells were seeded in 96‐well plates. After virus transfection or OGD/R treatment, CCK‐8 reagent was added to each well, and then the neurons were incubated for an additional 5 h, and the SH‐SY5Y cells were incubated for 2 h. Cell viability was determined by measuring the absorbance at 450 nm. The cell viability data are presented as percentages of the corresponding controls.

### 2.7 Adeno‐Associated Virus Construction and Stereotaxic Injection Methods

SFXN1 knockdown adeno‐associated virus was purchased from OBiO Technology (Shanghai, China) Corp. Ltd. To knock down SFXN1, the shRNA sequence: GAACGAACAGCTAGAGAAT was selected to construct the adeno‐associated virus vector (pAAV‐U6‐shRNA‐CMV‐EGFP‐WPRE). Viruses were stereotaxically injected into the cortex of mice at three sites (Site 1, 1.5 mm anterior to bregma, lateral 2.5 mm to midline, 2 mm deep; Site 2, 0.3 mm posterior to bregma, lateral 3 mm to midline, 2 mm deep; Site 3, 1.9 mm posterior to bregma, lateral 3 mm to midline, 2 mm deep) with a rate of 0.2 μL/min on the left. 1 μL (5 × 10^12^ vg/mL) of virus was slowly injected with a stainless‐steel cannula (10 μL Hamilton syringe) over 5 min for each site. At the end of the injection, the microinjector was kept in place for an additional 5 min before removal. After injection for 30 days, the left side of the mouse brain was subjected to tMCAO [39].

### 2.8 Lentivirus Infection of Neurons

SFXN1 knockdown lentivirus was purchased from OBiO Technology (Shanghai) Corp. Ltd. To knock down SFXN1, the shRNA sequence: GAACGAACAGCTAGAGAAT was used to construct the lentiviral vector GL427 (pSLenti‐U6‐shRNA‐CMV‐EGFP‐F2A‐Puro‐WPRE, Obio, Shanghai, China). The concentrated lentivirus was stably transfected into primary cortical neurons [43]. 3 days after the virus transfection, the neurons were used for further experiments.

### 2.9 Statistical Analysis

GraphPad Prism software was used for the statistical analysis. All the data are presented as the means ± SDs. The normality of the data was tested using the Shapiro–Wilk normality test. Data with a normal distribution were analyzed by Student's *t*‐test or ANOVA analysis. Student's *t*‐test was used for comparisons between two independent groups. One‐way or two‐way ANOVA with the Bonferroni correction or Tukey's test was used for multiple comparisons. The Kruskal–Wallis test was used to compare multiple groups that are not normally distributed. Statistical significance was set at *p* < 0.05.

## 3 Results

### 3.1 SFXN1 Expression Is Upregulated After Cerebral Ischemia–Reperfusion Injury

**FIGURE 1:**
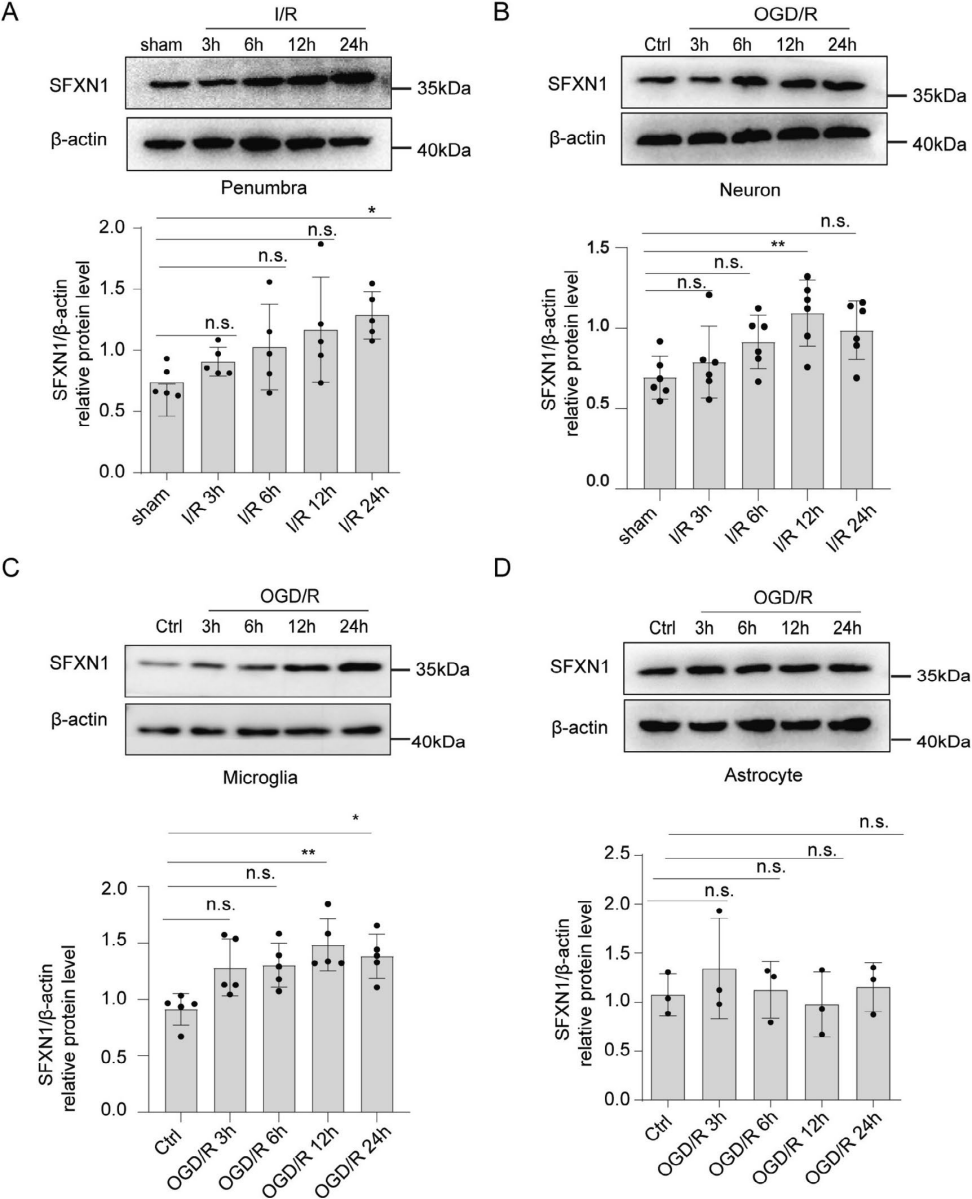
SFXN1 expression is upregulated after cerebral ischemia–reperfusion injury. (A) Representative immunoblot and relative intensity quantification of SFXN1 in mouse brain penumbra extracts after tMCAO at different time points of reperfusion, *n* = 5. (B–D) Representative immunoblot and quantification of SFXN1 in primary cultured cortical neurons (B, *n* = 6), primary cultured microglia (C, *n* = 5) and primary cultured astrocytes (D, *n* = 3) after OGD/R treatment for 1.5 h and followed by various time points of reoxygenation. The data are means ± SD for all panels: *p < 0.05, **p < 0.01, ***p < 0.001, n.s., no significance. One‐way ANOVA analysis (A–C) was used followed by Tukey test. Kruskal–Wallis test was used for (D). All data are representative of or combined from at least three independent experiments.

To examine the role of SFXN1 in I/R‐induced neuronal death, we first examined the expression pattern of SFXN1 in the brain following cerebral I/R. The expression of SFXN1 gradually increased in the penumbra of the cerebral cortex after tMCAO surgery in mice, whereas no significant change was observed in the contralateral region (Figure 1A and Figure S1A,B). To determine the cell types responsible for the increased expression of SFXN1, we cultured primary cortical neurons, microglia, and astrocytes, and assessed SFXN1 expression after OGD/reoxygenation (OGD/R). SFXN1 expression was significantly upregulated in cortical neurons after OGD/R, which is consistent with its expression pattern in the penumbra after tMCAO (Figure 1B). Similarly, primary microglia also presented increased SFXN1 expression after OGD/R (Figure 1C and Figure S2A). In contrast, SFXN1 expression in primary astrocytes remained unchanged, as confirmed by Western blot and immunofluorescence staining (Figure 1D and Figure S2B). These results suggest that SFXN1 expression is upregulated in neurons and microglia following cerebral I/R injury.

### 3.2 SFXN1 Knockdown Reduces OGD/R‐Induced Neuronal Death

**FIGURE 2:**
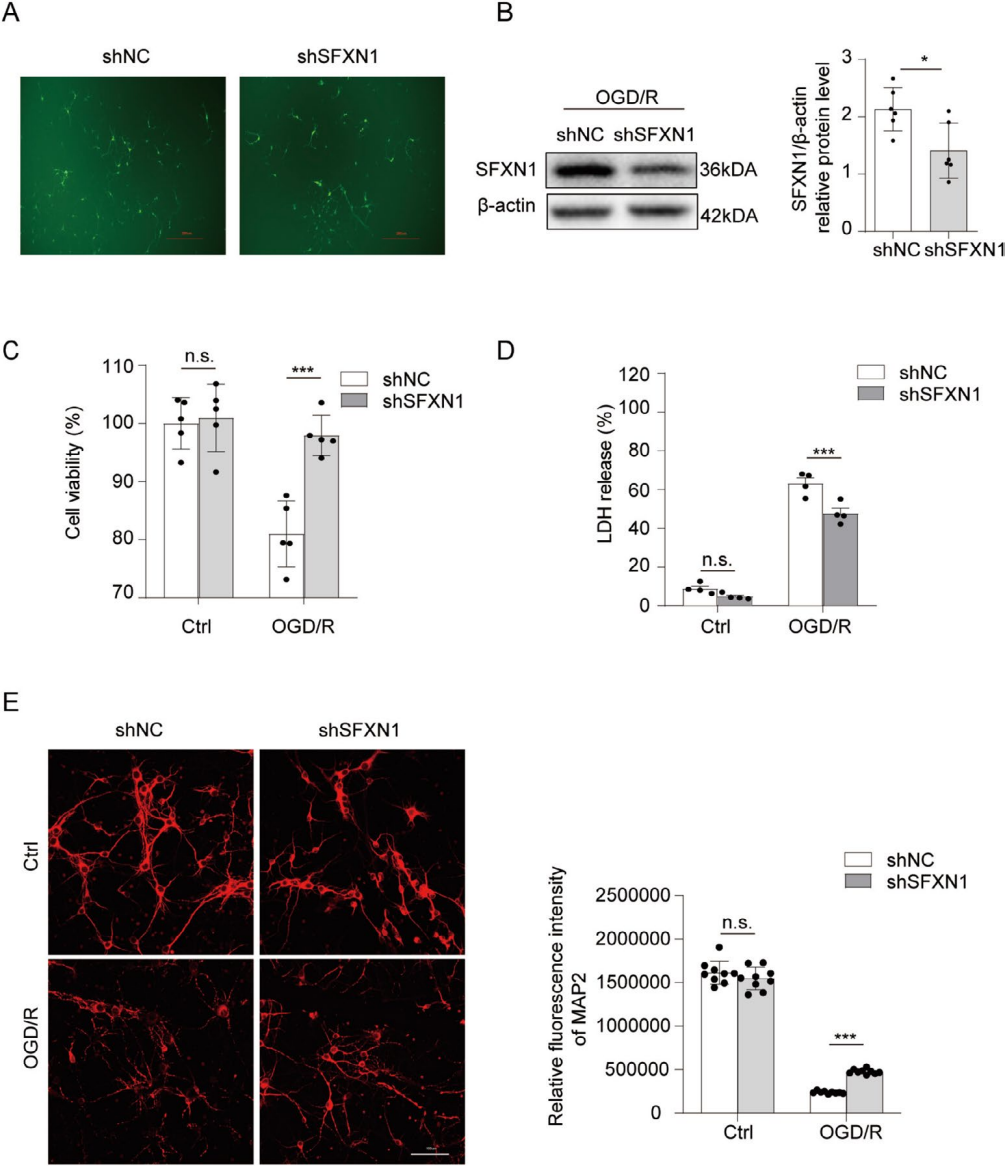
Knockdown of SFXN1 reduced OGD/R‐induced neuronal death. (A) The representative transfection efficiency of the GFP‐containing lentivirus in the cultured cortical neurons for 3 days. Scale bar, 250 μm. (B) Immunoblot and quantification of SFXN1 intensity in primary neurons transfected with indicated lentiviruses for 3 days. β‐actin was used as a loading control, *n* = 6. (C, D) The primary cultured cortical neurons were first transfected with shNC and shSFXN1 lentiviruses for 3 days and then subject to OGD treatment for 1.5 h and reoxygenation for 24 h. Cell viability was evaluated for (C, *n* = 5), and LDH release was tested for (D, *n* = 4). (E) Representative MAP2 immunofluorescence staining and statistical analysis in primary cortical neurons infected with shNC and shSFXN1 lentivirus for 3 days followed by OGD treatment for 1.5 h and reperfusion for 24 h. Scale bar, 100 μm, *n* = 3. The data are means ± SD for all panels: *p < 0.05, ***p < 0.001, n.s., no significance. Student's *t*‐test (B) was used, Two‐way ANOVA analysis (C–E) was used followed by Bonferroni's comparison test.

To determine whether SFXN1 directly affects neuronal death after OGD/R, we used lentivirus to knock down SFXN1 and assess its impact on neuronal viability. Immunofluorescence staining confirmed the efficient transfection of primary cultured cortical neurons with both shNC and shSFXN1 lentiviruses after 72 h (Figure 2A). Western blot analysis revealed that, compared with shNC‐transfected primary cultured neurons, shSFXN1‐transfected neurons showed reduced SFXN1 expression (Figure 2B). SFXN1 knockdown increased neuronal resistance to OGD/R‐induced cell death as evidenced by increased cell viability observed in the CCK8 assay, reduced LDH release, and increased MAP2 intensity (Figure 2C–E). In addition, the knockdown of SFXN1 in SH‐SY5Y cells [44] (a neuroblastoma cell line widely used cell lines in neurosciences) resulted in increased cell viability and decreased LDH release (Figure S3A–C). These findings indicate that SFXN1 knockdown reduces neuronal death after OGD/R.

### 3.3 SFXN1 Knockdown Improves Mitochondrial Function After OGD/R

**FIGURE 3:**
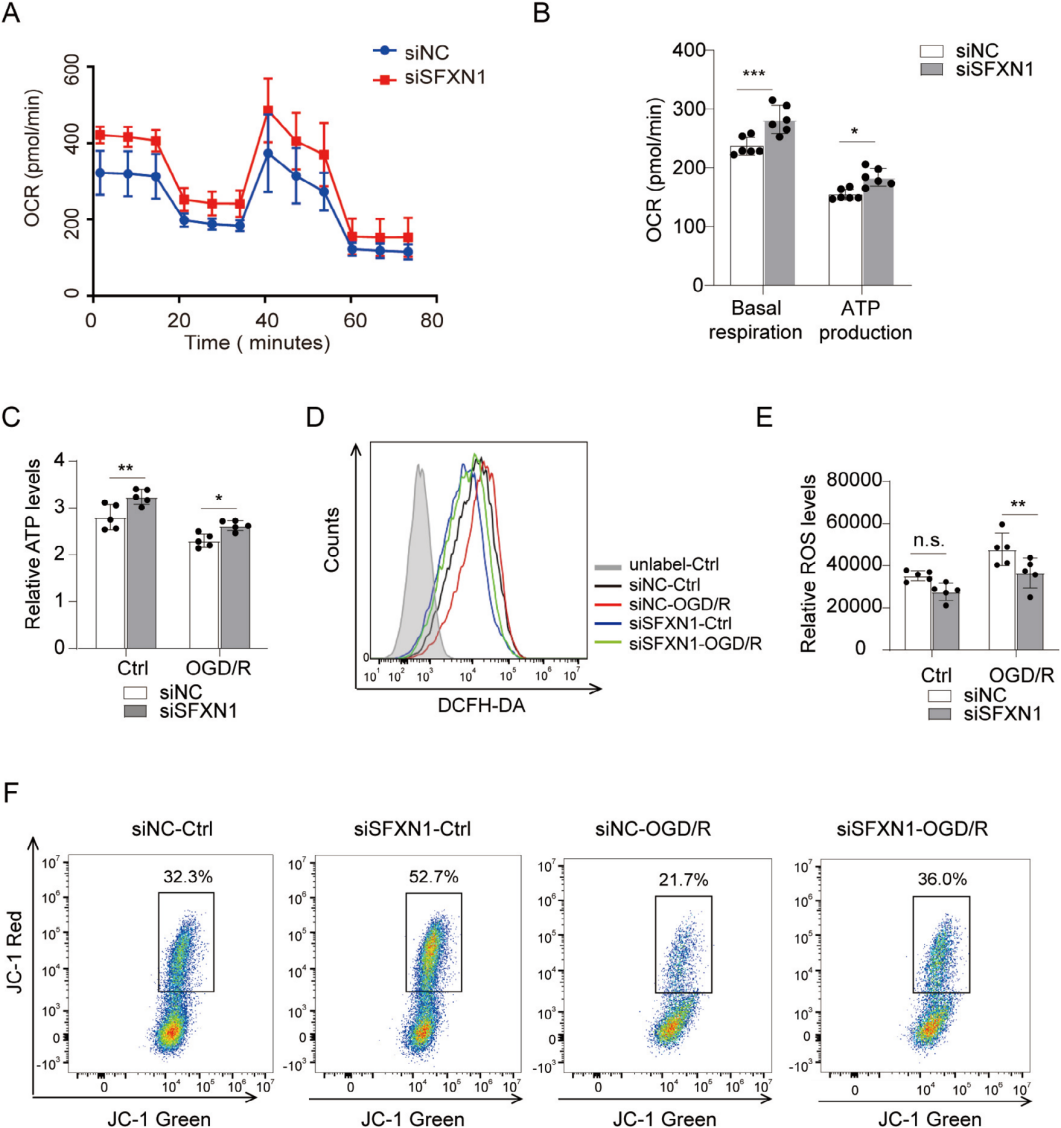
Knockdown of SFXN1 improved mitochondrial function of SH‐SY5Y cells after OGD/R. (A) Representative OCR analysis of siNC and siSFXN1‐transfected SH‐SY5Y cells after OGD/R treatment. (B) Statistical graphs of basal respiration and ATP production. (C) ATP release was assessed in SH‐SY5Y cells transfected with siNC or siSFXN1 for 48 h, and subjected to OGD treatment for 2 h and reperfusion for 12 h, *n* = 6. (D, E) Flow cytometry analysis and quantification of DCFH‐DA in SH‐SY5Y cells transfected with siNC or siSFXN1 for 48 h and subjected to OGD treatment for 4 h and reperfusion for 12 h, *n* = 5. (F) Flow cytometry analysis of mitochondrial membrane potential with JC‐1 probes in SH‐SY5Y cells transfected with siNC or siSFXN1 for 48 h and subjected to OGD treatment for 4 h and reperfusion for 12 h. The data are means ± SD for all panels: *p < 0.05, **p < 0.01, ***p < 0.001, n.s., no significance. Two‐way ANOVA analysis (B, C, E) was used followed by Bonferroni's comparison test.

Given that SFXN1 is a serine and iron transporter located on the inner mitochondrial membrane, we investigated whether SFXN1 protects neurons against OGD/R‐induced cell death by modulating mitochondrial function, as mitochondrial dysfunction is a key contributor to I/R injury [45, 46]. Immunofluorescence staining confirmed that SFXN1 was located mainly in the mitochondria of primary cultured cortical neurons (Figure S2C). Given the central role of mitochondria in aerobic respiration, we employed oxygen consumption rate (OCR) assays to assess mitochondrial respiratory function in SFXN1‐knockdown SH‐SY5Y cells. Compared with control cells, SFXN1‐knockdown presented significantly elevated mitochondrial respiratory parameters, including basal respiration and ATP production, after OGD/R (Figure 3A,B). Since mitochondria are the primary sites of energy production [47], we measured intracellular ATP levels after SFXN1 knockdown. Compared with the control group, the OGD/R group presented lower intracellular ATP levels, whereas SFXN1‐knockdown neurons presented higher ATP levels (Figure 3C). Reactive oxygen species (ROS) production, another important aspect of mitochondrial dysfunction, was also assessed. Using a DCFH‐DA sensor, we found that SFXN1 knockdown in SH‐SY5Y cells significantly reduced the ROS levels induced by oxygen–glucose deprivation/reoxygenation (OGD/R) compared with those in control cells (Figure 3D,E). Additionally, mitochondrial electric potential, another indicator of mitochondrial function, was decreased in control SH‐SY5Y cells after OGD/R but was restored to near‐normal levels in SFXN1‐knockdown cells, as demonstrated by JC‐1 staining (Figure 3F). These results suggest that SFXN1 knockdown improved mitochondrial function in neurons and neuronal cell lines after OGD/R.

Given that SFXN1‐mediated serine transport is associated with reduced ROS levels [48] and that iron transport results in increased mitochondrial ROS [25], we explored whether the reduction in SFXN1 promoted neuronal survival in an iron transport‐dependent manner. Treatment with deferoxamine B (DFO), an iron chelator [49, 50, 51], did not abrogate the increased viability of SFXN1‐knockdown SH‐SY5Y cells after OGD/R (Figure S4A). Consistently, no significant difference in lipid peroxidation was detected by C11‐BODIPY 581/591 staining, revealing no significant difference between the SFXN1‐knockdown SH‐SY5Y cells and the control cells (Figure S4B,C). Therefore, SFXN1 promotes neuronal death after OGD/R in an iron‐independent manner.

### 3.4 SFXN1 Knockdown Reduces Inflammatory Cytokine Production in Microglia

**FIGURE 4:**
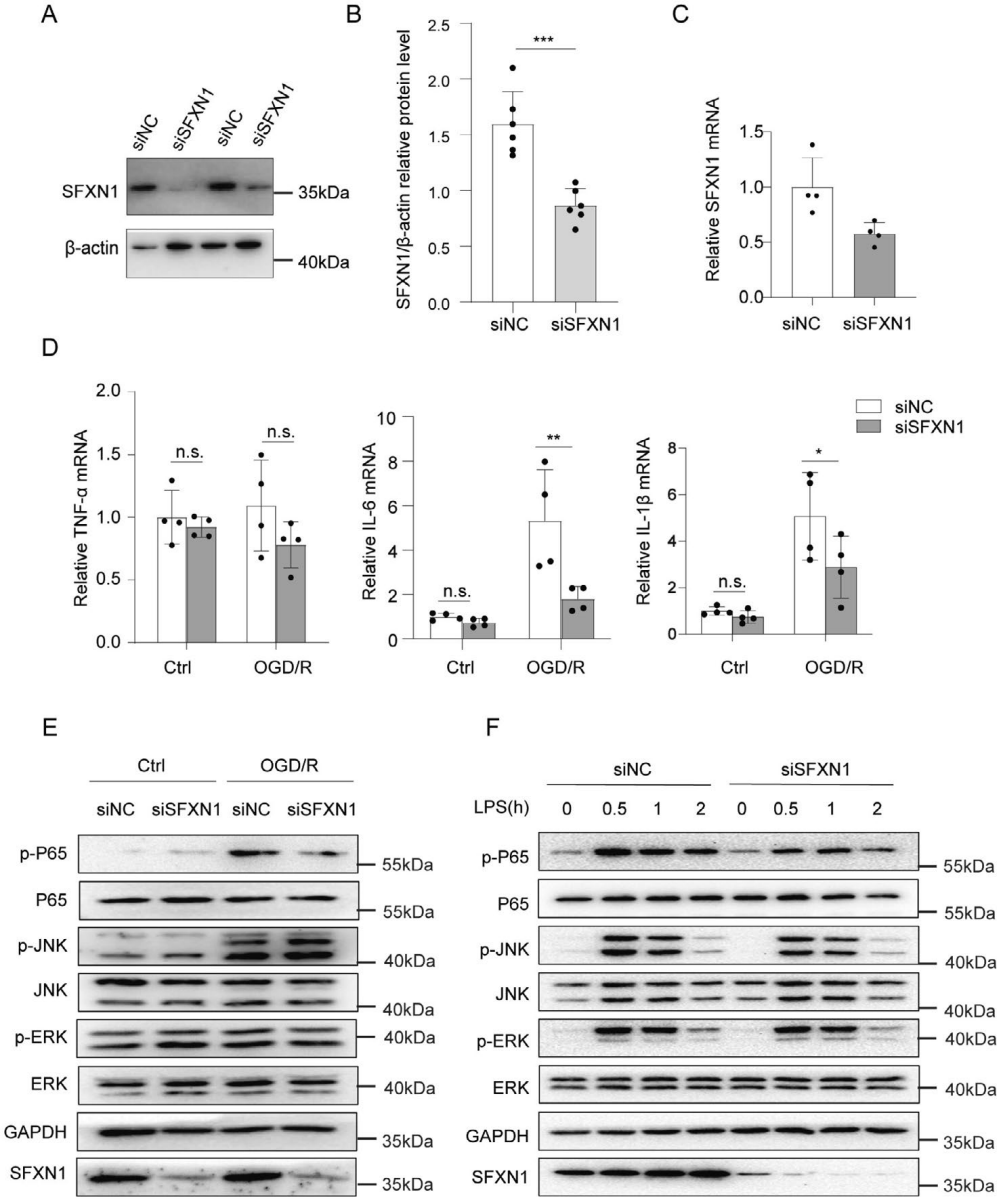
SFXN1 knockdown inhibits the expression of proinflammatory cytokines in microglial cells. (A) Representative immunoblot and (B) quantification of SFXN1 in BV2 microglial cells transfected with siNC or siSFXN1 for 48 h, *n* = 6. (C) The mRNA level of SFXN1 in BV2 microglial cells transfected with siNC or siSFXN1, *n* = 4. (D) The qRT‐PCR analysis of cytokines (TNF‐α, IL‐1β, and IL‐6) mRNA levels in siNC or siSFXN1‐transfected BV2 microglial cells after OGD treatment for 2 h and reperfusion for 12 h, *n* = 4. (E) Representative immunoblot of total or phosphorylated (p‐) proteins in BV2 microglial cells transfected with siNC or siSFXN1 after OGD treatment for 2 h and reperfusion for 6 h, *n* = 3. (F) Representative immunoblot of total and phosphorylated (p‐) proteins of lysates from LPS‐stimulated or PBS‐stimulated BV2 microglial cells after transfection with siNC or siSFXN1, *n* = 3. The data are means ± SD, for all panels: *p < 0.05, **p < 0.01, ***p < 0.001 by Student's *t* test (B, C) and Two‐Way ANOVA analysis (D) followed by Sidak's multiple comparisons test. All data are representative of or combined from at least three independent experiments.

Considering the significant upregulation of SFXN1 in microglia after OGD/R, we investigated whether SFXN1 plays a role in the inflammatory response of microglia. SFXN1 was successfully knocked down in BV2 microglia using small interfering RNA (siRNA) targeting SFXN1 (Figure 4A–C). SFXN1 knockdown reduced the mRNA levels of IL‐6 and IL‐1β in BV2 microglia after OGD/R compared with those in siNC‐transfected cells, whereas the TNF‐α level remained unchanged (Figure 4D). Given that astrocytes are also involved in neuroinflammatory processes, we further investigated the role of SFXN1 in OGD/R‐induced inflammation in astrocytes, although SFXN1 was not upregulated in astrocytes following OGD/R treatment. Compared with those in siNC‐transfected cells, the mRNA levels of IL‐6, IL‐1β, and TNF‐α in primary astrocytes remained unchanged after knocking down SFXN1 (Figure S5A,B).

As lipopolysaccharide (LPS) stimulation induces a robust inflammatory response in microglia and activates signaling pathways similar to those triggered by OGD/R, we further examined the effects of SFXN1 on inflammatory cytokine expression in BV2 microglia upon LPS stimulation. Similarly, compared with siNC, the two siRNAs decreased the mRNA levels of IL‐6 and IL‐1β in LPS‐stimulated BV2 microglia (Figure S5C–E). Therefore, SFXN1 knockdown leads to the reduced production of inflammatory cytokines in microglia.

### 3.5 SFXN1 Promotes NF‐κB Signaling Activation Partially Through Iron Transport

To explore the molecular mechanism by which SFXN1 promotes proinflammatory cytokine expression in microglia, we examined the activation of downstream signaling pathways, including the extracellular regulated protein kinase (ERK), nuclear factor kappa light chain enhancer of activated B cells (NF‐κB), and c‐Jun N‐terminal kinase (JNK) pathways, which are well‐established signaling cascades activated by OGD/R or LPS stimulation [52]. Notably, the phosphorylation of the NF‐κB subunit P65 induced by OGD/R was significantly reduced in BV2 microglia after SFXN1 was knocked down, whereas the phosphorylation of JNK and ERK remained unchanged (Figure 4E). Moreover, we verified the effects of SFXN1 on inflammatory signal transduction in microglia after LPS stimulation. Similarly, in LPS‐stimulated BV2 microglia, SFXN1 knockdown suppressed the phosphorylation of the NF‐κB subunit P65 without affecting ERK and JNK activation (Figure 4F). Thus, knocking down SFXN1 suppresses proinflammatory cytokine expression in microglia by attenuating the activation of NF‐κB signaling.

Given the reduced ROS levels observed in SFXN1‐knockdown SH‐SY5Y cells, we investigated whether SFXN1 regulates inflammatory cytokine expression by modulating ROS levels in microglia. However, SFXN1 knockdown did not alter ROS levels in LPS‐stimulated BV2 microglia (Figure S6A,B). Since SFXN1 mediates iron transport from the cytoplasm to the mitochondria, we further determined whether iron homeostasis contributes to SFXN1‐mediated inflammatory cytokine production. Notably, the reduced expression of proinflammatory cytokines in SFXN1‐knockdown BV2 microglia was partially restored after treatment with DFO (Figure S6C). Consistently, DFO treatment also restored NF‐κB activation in SFXN1‐knockdown BV2 microglia (Figure S6D). These results suggest that SFXN1 may promote proinflammatory cytokine production by promoting NF‐κB signaling activation partially through iron transport.

### 3.6 SFXN1 Knockdown Suppresses Inflammatory Response and Reduces Neurotoxicity

**FIGURE 5:**
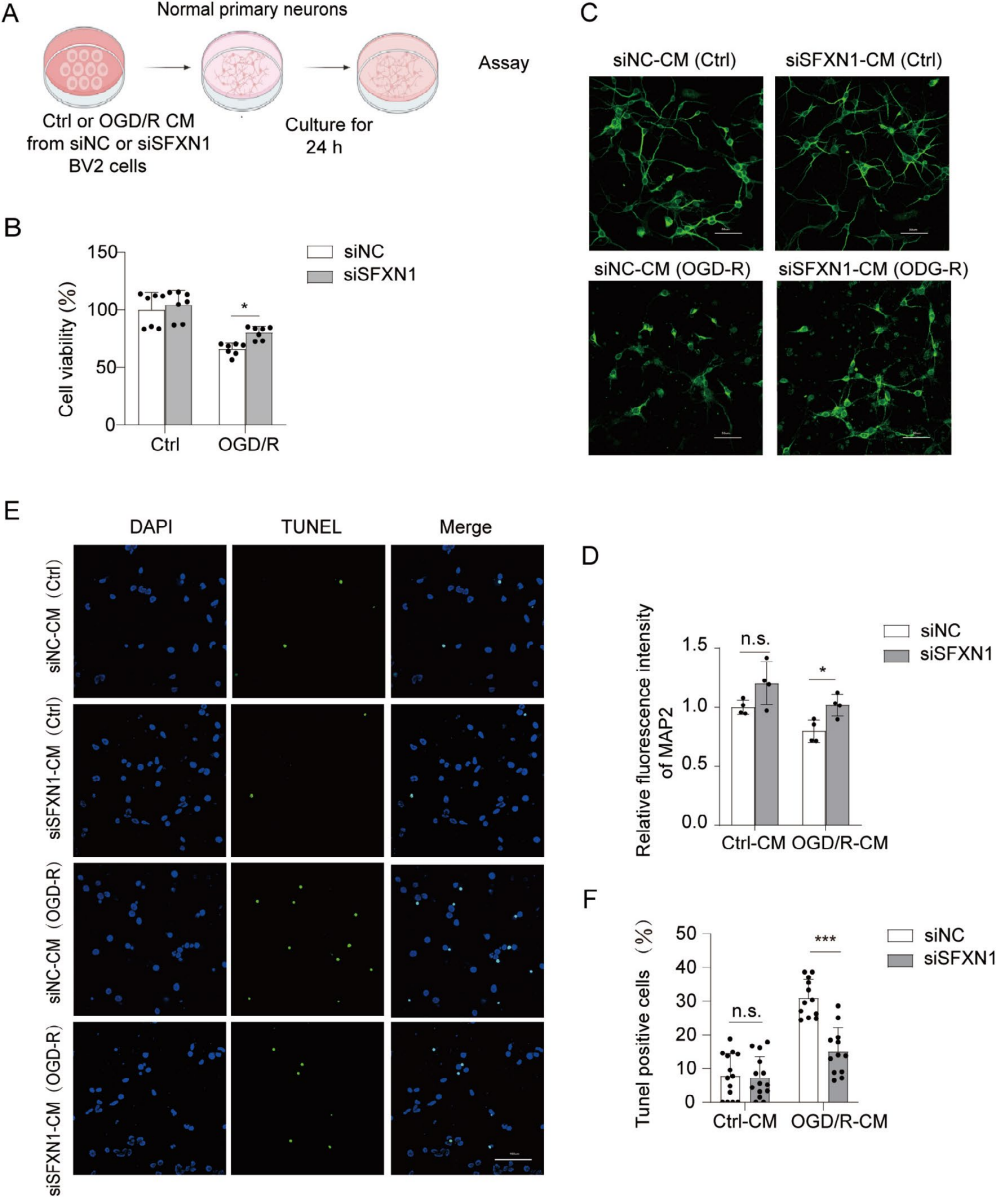
SFXN1‐knockdown microglial cells exhibit reduced neurotoxicity in vitro. (A) Diagram for the co‐culture of BV2 cell‐conditioned medium with neurons. (B) CCK8 assay of neurons co‐culturing with conditioned medium (CM) from siNC or siSFXN1‐transfected BV2 microglial cells that subjected to OGD/R treatment, *n* = 7. (C) Representative MAP2 immunofluorescence staining in primary cortical neurons co‐culturing with conditioned medium from siNC or siSFXN1‐transfected BV2 microglial cells. Scale bar, 50 μm. (D) Quantitative analysis of MAP2 intensity to assess neuronal viability using relative fluorescence intensity, *n* = 4. (E) TUNEL staining and (F) quantitative analysis of TUNEL^+^ neurons cocultured with CMs from siNC or siSFXN1‐transfected BV2 microglia cells after OGD/R treatment, *n* = 3. Scale bar, 100 μm. The data are means ± SD, for all panels: n.s., no significance, **p < 0.01, ***p < 0.001 by Two‐Way ANOVA analysis followed by Bonferroni Test (B, D) and Sidak's test (F).

After ischemic stroke, cytokines released by microglia are generally considered toxic to neurons [53]. To directly assess the impact of reduced microglial activation due to a reduction in SFXN1 expression on neuronal viability, we cultured neurons with conditioned medium from OGD/R‐treated SFXN1‐knockdown or siNC‐transfected BV2 microglia (Figure 5A). As predicted, conditioned medium from OGD/R‐treated BV2 microglia reduced neuronal viability compared with conditioned medium from untreated BV2 microglia. However, conditioned medium from SFXN1‐knockdown BV2 microglia was less neurotoxic than that from the corresponding controls, as evidenced by the CCK8 assay (Figure 5B). Consistent with these results, MAP2 staining and TUNEL staining also demonstrated reduced neurotoxicity with conditioned medium from SFXN1‐knockdown BV2 microglia compared with that from control microglia (Figure 5C–F). Thus, SFXN1 knockdown in microglia results in decreased neurotoxicity in vitro.

### 3.7 SFXN1 Knockdown Protects Mice Against Cerebral I/R Injury

**FIGURE 6:**
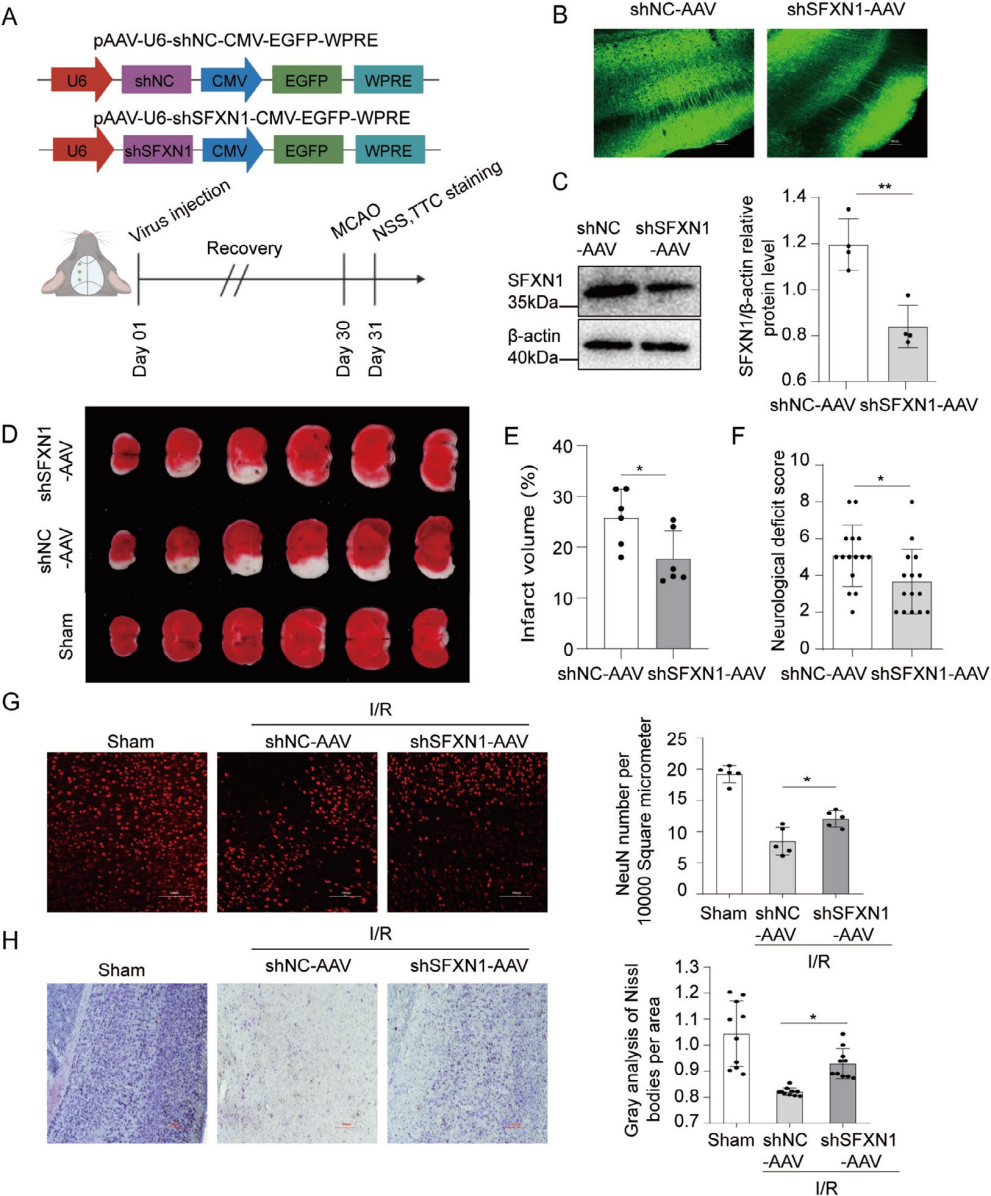
Knockdown of SFXN1 alleviates ischemic brain injury. (A) Schematic diagram of lentivirus stereotaxic injection, tMCAO, NSS analysis and TTC staining schedule. (B) Representative image showing typical infection efficiency of AAV (GFP) in cortex of the brain. Scale bar, 100 μm, *n* = 6. (C) Representative immunoblot and quantification of SFXN1 in corresponding mouse brain tissues after tMCAO after injection with shNC‐AAV or shSFXN1‐AAV for 4 weeks. β‐actin served as a loading control, *n* = 3. (D) Brain infarction stained by TTC at 24 h after tMCAO in shNC‐AAV and shSFXN1‐AAV injected mice. (E) Quantification of brain infarct volume at 24 h after reperfusion. *n* = 6 mice per group. (F) Neurological severity score at 24 h after reperfusion, *n* = 15 mice per group. (G) Representative brain slices in different groups stained with anti‐NeuN antibodies to label neurons (left) and quantification of relative number of neurons at 24 h after reperfusion. The view shown located in the infarct penumbra, *n* = 5 mice per group. Scale bar, 100 μm. AU, arbitrary units. (H) Representative Nissl staining image and gray analysis of infarct penumbra at 24 h after reperfusion, *n* = 5 mice per group. Scale bar, 500 μm. The data are means ± SD, for all panels: *p < 0.05, **p < 0.01 by Student's *t* test (C, E, F). One‐way ANOVA analysis (G, H) was used followed by Tukey test.

Considering the important role of SFXN1 in regulating neuronal survival and microglial inflammation after OGD/R, we investigated whether SFXN1 knockdown alleviates cerebral I/R injury in vivo. We used adeno‐associated virus (AAV) to knock down SFXN1 in C57BL/6 mice and then performed tMCAO to analyze ischemic brain injury (Figure 6A). The AAV‐shNC and AAV‐shSFXN1 viruses presented high transfection efficiency in cortical brain cells, especially neurons (Figure 6B and Figure S7). The abundance of SFXN1 was lower in brains injected with the AAV‐shSFXN1 virus than in those injected with the AAV‐shNC virus (Figure 6C). Notably, the AAV‐shSFXN1‐injected brains presented a smaller infarct volume than did the brains injected with the AAV‐shNC virus (Figure 6D,E). Accordingly, reducing the expression of SFXN1 also decreased the neurological deficit score after tMCAO compared with that in the corresponding control groups (Figure 6F). Therefore, reducing SFXN1 expression alleviated ischemic brain injury.

To further confirm the protective effect of SFXN1 knockdown on neurons, we performed NeuN staining and Nissl staining. NeuN immunofluorescence staining revealed that compared with that in shNC‐AAV‐injected mice, the number of SFXN1‐knockdown neurons was greater in mice after I/R injury (Figure 6G). Nissl staining revealed that neurons in the sham control mice had more regularly shaped Nissl bodies, whereas neurons in AAV‐shNC‐injected mice exhibited fewer and deformed Nissl bodies after I/R injury than did neurons in AAV‐shSFXN1‐injected mice. These pathological changes were alleviated in the brains of AAV‐shSFXN1‐injected mice (Figure 6H). These data suggest that SFXN1 knockdown alleviated neuronal death after cerebral I/R injury.

## 4 Discussion

SFXN1 is a mitochondrial inner membrane protein that is involved primarily in the transport of iron and serine. However, the role of SFXN1 in ischemic stroke remains unknown. In this study, we found that SFXN1 expression was upregulated after cerebral I/R and that knocking down SFXN1 in vivo reduced the brain infarct volume following ischemic stroke. Mechanistically, SFXN1 can induce mitochondrial dysfunction to reduce neuronal survival after OGD/R in an iron‐independent manner and promote proinflammatory cytokine production in microglia by activating NF‐κB signaling partially through iron transport after OGD/R.

Mitochondria are vital to neuronal survival under both physiological and pathological conditions [54, 55]. Various aspects of mitochondrial function, such as ATP production, mitochondrial membrane potential depolarization, and ROS production have been implicated in neuronal death after ischemic stroke [56, 57]. Mitochondrial carriers facilitate the transport of metabolites between the cytoplasm and mitochondria, enabling metabolic flexibility and plasticity to maintain homeostasis in response to changing metabolic demands [58]. However, how these metabolites affect neuronal survival after ischemic stroke remains poorly understood. In this study, we identified SFXN1, a mitochondrial metabolite transporter primarily involved in iron and serine transport, as a mediator of neuronal death after cerebral I/R injury. SFXN1 knockdown in neuronal cell lines enhanced mitochondrial function, as characterized by increased ATP production, decreased ROS levels, and increased mitochondrial membrane potential. Previous studies have shown that SFXN1 can increase mitochondrial ROS levels by promoting iron transport from the cytoplasm to the mitochondria [59]. Moreover, SFXN1‐mediated amino acid transport can also facilitate mitochondrial function by regulating mitochondrial integrity and one‐carbon metabolism in HEK293T cells [23, 24]. In this study, we found that treatment with an iron chelator failed to reverse the increase in neuronal viability observed after SFXN1 knockdown, suggesting that iron transport is not involved in SFXN1‐mediated neuronal death, although whether serine transport is involved in this process was not investigated due to technical limitations. These findings reveal that SFXN1 may play different roles in different cells, which is probably due to its preference for transporting metabolites, and this issue still needs further investigation.

Previous studies have demonstrated that SFXN1 is involved in various physiological and pathological processes. For example, SFXN1 is required for erythroid cell development, and SFXN1 knockout leads to hypochromic anemia in zebrafish [21]. Additionally, SFXN1 upregulation has been observed in certain types of cancers, such as lung adenocarcinoma and triple‐negative breast cancer, and is associated with poor prognosis [60, 61, 62]. As a mitochondrion‐localized protein, SFXN1 also plays a role in tissue injury. SFXN1 has been implicated in sepsis‐induced cardiac injury [59] and acute lung injury [63] by modulating ferritinophagy‐mediated ferroptosis. Moreover, SFXN1‐mediated mitochondrial iron overload regulates fibrosis and cardiomyocyte hypertrophy [25, 64]. In this study, we revealed the involvement of SFXN1 in ischemia–reperfusion brain injury, highlighting its multifaceted roles in both development and disease progression.

Neuroinflammation is a critical factor in the pathophysiology of ischemic stroke [65, 66, 67]. Both microglia and astrocytes can be activated after brain injury and play key roles in regulating the progression of brain injury [68]. In this study, we found that the expression of SFXN1 was increased in microglia but unchanged in astrocytes after OGD/R. However, owing to limitations associated with the selected antibody in our study, we were unable to assess SFXN1 expression in brain tissues. SFXN1 knockdown in microglia reduced the expression of proinflammatory cytokines (TNF‐α, IL‐6, and IL‐1β) and suppressed NF‐κB‐P65 activation following both LPS stimulation and OGD/R. However, cytokine expression was not altered in astrocytes after knocking down SFXN1, indicating the different roles of mitochondrial SFXN1 in the inflammatory response of microglia and astrocytes subjected to OGD/R. Unlike those in neuronal cells, ROS levels were not altered in SFXN1‐knockdown microglia. Moreover, the iron chelator deferoxamine (DFO) partially abrogated the reduction in proinflammatory cytokine production in SFXN1‐knockdown BV2 microglia but did not affect the increased viability of SH‐SY5Y cells following SFXN1 knockdown. These findings suggest that SFXN1‐mediated iron transport has distinct roles in regulating cell survival and inflammatory responses. For cell survival, metabolites affecting energy production or protein synthesis may be more critical, whereas for inflammatory responses, metabolites that trigger inflammatory signaling pathways are likely more important. During inflammation, cytoplasmic iron accumulation may influence inflammatory cytokine signaling, whereas in OGD/R‐induced cell death, multiple metabolic pathways and metabolites are significantly altered. Additionally, other iron transporters or adaptors may compensate for the effects of SFXN1 on cell death.

Our study has several limitations. In this study, we used a well‐established in vitro OGD/R system to investigate the role of SFXN1 in neuronal survival and microglia‐mediated neuroinflammation. To examine the effects of SFXN1 on ischemic stroke, we injected AAVs into the cortex of mice to knock down SFXN1 and assessed its impact on the brain infarct volume after ischemic stroke. However, since AAVs mainly infect neurons rather than microglia [69, 70], and no highly effective virus currently exists that can infect both cell types simultaneously, we were unable to evaluate the role of SFXN in microglia‐mediated neuroinflammation in vivo. The generation of conditional knockout mice may help address this limitation in the future. In addition, in vitro coculture experiments demonstrated that conditioned medium from SFXN1‐knockdown microglia reduced neurotoxicity, indicating the protective effect of SFXN1 knockdown against neuroinflammation and neuronal injury.

## 5 Conclusion

In conclusion, our study reveals the neuroprotective effects of reducing SFXN1 expression after cerebral I/R injury, which is achieved by promoting neuronal survival and suppressing microglia‐mediated neuroinflammation. These findings suggest that SFXN1 may serve as a potential therapeutic target for ischemic stroke.

## Author Contributions

X.X., Z.D., and X.Z. designed and performed most experiments, analyzed data, and prepared the manuscript; R.Z. and Z.Z. performed all the MCAO and analyzed data; J.X. and M.L. helped with the WB and IF experiments; Q.W. and Y.C. conceptualized the research, directed the study, and prepared the manuscript.

## Ethics Statement

All animal experiments were conducted in compliance with National Institutes of Health guidelines and were approved by the institutional animal care and use committee of Qingdao University (No. 201905C57180202208049).

## Conflicts of Interest

The authors declare no conflicts of interest.

## Supporting information


Data S1.

## Data Availability

All relevant data are within the paper and its Supporting Information.
