# Supplementary material for: SFXN1 Reduction Alleviates Cerebral Ischemia–Reperfusion Injury by Promoting Neuronal Survival and Reducing Neuroinflammation
Source: CNS Neurosci Ther. 2025 May 26;31(5):e70457. doi: 10.1111/cns.70457 (PMC12106369; doi:10.1111/cns.70457)

Supplementary Information

Supplementary Materials and Methods

Neurological severity score (NSS) analysis

At 24 h after tMCAO, the mice were evaluated using the following 9-point scoring system according to previous reports[1, 2]. 0, normal neurological function; 1, left forelimb flexion upon held by the tail or failure to extend the right forepaw fully; 2, left shoulder adduction after tail suspension; 3, decreased resistance to a lateral push toward the left; 4, spontaneous movement in all directions, with circling to the left only if pulled by the tail; 5, walking or circling spontaneously to the left; 6, walking only when stimulated; 7, no reaction to stimulation; 8, stroke-induced death.

Antibodies and reagents

The following antibodies were purchased from Cell Signaling Technology: Phospho-NF-κB p65 (Ser536) (93H1) (3033S, rabbit), NF-kappa-B p65 (C22B4) (4764S, rabbit), Phospho-SAPK/JNK (Thr183/ Tyr185) (81E11) (4668, rabbit), SAPK/JNK Antibody (9252), p44/42 MAPK (Erk1/2) Antibody (4695) and Phospho-p44/42 MAPK (Erk1/2) (Thr202/Tyr204) Antibody (9101S). β-actin (bs-0061R) Ab was purchased from Proteintech. SFXN1(HPA019543) was purchased from Atlas, SFXN1 (A12954) was purchased from Abclonal. NeuN (ab177487) was purchased from Abcam. Goat anti-rabbit IgG-HRP and Goat anti-mouse IgG-HRP were purchased from KeRui Biotechnology (China). Goat anti-mouse IgG-AlexaFluor-488 conjugate (bs-0296G) was purchased from Bioss. GFAP (Z0334) was purchased from DAKO. IBA-1 (019-19741) was purchased from Wako Chemical. MAP2 (M4403) was purchased from

Sigma. DAPI (S2110) was purchased from Solarbio. Reactive Oxygen Species Assay Kit (S0033S), ATP Assay Kit (S0026) and Mitochondrial membrane potential assay kit with JC-1 (C2006) were purchased from Beyotime. LPS (L2630) was purchased from Sigma. Nissl Staining Solution (C0117) was from Beyotime.

Cell culture

The SH-SY5Y cell line was purchased from the National Collection of Authenticated Cell Cultures (Shanghai, China). BV2 cell line was obtained from Cell Resource Center, Institute of Basic Medical Science, Chinese Academy of Medical Sciences (Beijing, China). These cell lines were cultured in DMEM (Solarbio, China) containing 10 % fetal bovine serum (Gibco, USA) and 1 % penicillin/ streptomycin (Gibco, USA).

Cortical neurons were prepared from embryonic-day-17 brains of female mice (C57BL/6) as described previously[3, 4]. Briefly, cortex of the embryos was harvested and digested with 0.05 % trypsin to prepare single-cell suspensions. The dissociated cortical neurons were suspended in corresponding plating medium (Neurobasal medium, 2 % B-27 supplement, 0.5 mM L-glutamine and 25 mM glutamic acid) and seeded onto poly-D-lysine coated dishes or 48-well plate. After culturing for 1 day, half of the plating medium was renewed with maintenance medium (neurobasal medium, 0.5 mM L-glutamine, and 2 % B-27 supplement). Culture media were then changed every three days. The neurons were cultured for 11 days and then used for further experiments.

Primary microglia and astrocyte culture protocols were adapted from previous literatures[5, 6]. In brief, the neonatal brains from C57BL/6 mice (P1 or P2) were

removed and digested. Cells were then cultured in poly-D-lysine-coated six-well plates or 75 cm^2 flasks filled with DMEM/Ham's F12 medium (10 % FBS, penicillin, and streptomycin). Medium was renewed every three days. On day 9, the cells reached about 90 % confluence and were then digested with 0.05 % trypsin solution (0.05 % trypsin in DMEM/Ham's F12) at 37 °C for 30-40 min and removed detached cells. The obtained microglia were continued to culture until use. To isolate astrocytes, the cells were cultured for 9 to 12 days. When astrocytes were confluent and overlaying microglia were exposed on or had detached from the astrocyte layer, astrocytes were harvested by shaking the flask at 250 rpm for 16-18 hours to remove microglia and oligodendrocyte precursor cells. Discard the supernatants and wash the remaining astrocyte layer with PBS, add 0.25 % trypsin-EDTA and maintain at 37 °C for 5 min in the CO2 incubator. Add astrocytes culture medium when astrocytes were fully detached from the culture flask. The astrocytes were centrifuged at 1000 rpm for 5 min and fresh plating medium was added. The obtained astrocytes were tested for their purity by immunostaining and then were used for further experiments.

Immunoblot analysis

Immunoblot analysis was carried out as previously described [7]. Briefly, the protein samples were separated via SDS-PAGE after electrophoresis and transferred onto a polyvinylidene difluoride (PVDF) membrane. 5 % skim milk in Tris-buffered saline was used to block the PVDF membranes for 60 min at room temperature (RT). The PVDF membranes were then probed with the appropriate primary antibodies at 4 °C overnight. After washing with TBST buffer for three times, the membranes were

incubated with corresponding HRP-conjugated secondary antibodies at room temperature for 2 h. Protein bands were visualized by immunoblot analysis with the chemiluminescence detection system (Protein Simple or Image Quant 800, Cytiva, USA). Image J (Rawak Software, Inc, Germany) software was used to calculate band intensities.

Conditioned media (CM) assays

NC and SFXN1 siRNA were transfected into BV2, and OGD/R treatment was performed 48 h after transfection. The supernatants were harvested by centrifuge to remove cell debris. Primary cortical neurons were incubated with the conditioned medium (diluted with fresh medium at a 1:1 ratio) for 24 h. Neuronal viability was then measured.

Measurement of ATP content

Aspirate the culture medium, and add the lysate according to the ratio of adding 200 microliters of lysate to each well of a 6-well plate. First, add 100 microliters of ATP detection working solution to the detection well and incubate at room temperature for 3-5 minutes. Then add 20 microliters of sample or standard sample to the detection well, quickly mix with a micropipette, and detect with a chemiluminescence instrument after an interval of at least 2 seconds.

Intracellular ROS Measurement

The intracellular ROS levels were measured using a peroxide-sensitive fluorescent probe (DCFH-DA; Beyotime, China) following the manufacturer’s protocols. After

diluting DCFH-DA to a final concentration of 10 μM, the cell culture medium was removed, and an appropriate amount of diluted DCFH-DA was added and incubated with the cells for 20 min at 37 °C [8]. After incubation, the cells were collected and washed twice with PBS. Cell fluorescence was acquired on a flow cytometer (BECKMAN CytoFLEX S) and analyzed with software (FlowJo).

Mitochondrial membrane potential Measurement

Mitochondrial membrane potential (ΔΨm) which was measured by using a JC-1 fluorescent probe (Beyotime, China). Briefly, cells under different conditions were washed with PBS and incubated with an appropriate volume of JC-1 working buffer for 20 min at 37 °C. After incubation, the cells were then collected and washed twice with PBS. Cell fluorescence was acquired on a flow cytometer (BECKMAN CytoFLEX S) and analyzed with software (FlowJo).

Quantitative real-time PCR (qRT-PCR) analysis

RNAfast200 purification kit (Fastagen, China) was used to extract total RNA of cultured cells. To obtain cDNA, the ReverTra Ace® qPCR RT Master Mix with gDNA Remover (TOYOBO) was utilized. Real-time PCR was then performed using 2×RealStar Green Power Mixture (GenStar, China) with the Biorad 96 touch machine (Biorad, USA). GAPDH or β-actin was used as the internal control. The primer sequences for qPCR were listed below: mouse SFXN1 forward primer:5′-TTTCTGGCAGTGGATAAACCAGTCC-3′; reverse primer: 5′-AGTTAGCAGCAG CTACAGCAGCGA-3′; mouse GAPDH forward primer : 5′-TGGTGAAGGTCGG TGTGAACGG-3′; reverse primer: 5′-ACTGTGCCGTTGAATTTGCCG-3′; mouse

TNFA forward primer: 5′-TGATCGGTCCCCAAAGGGATG-3′; reverse primer: 5′-TTGGTGGTTTGCTACGACGTGG-3′; mouse IL-6 forward primer: 5′-TGATGCA CTTGCAGAAAACAATCTGA-3′; reverse primer: 5′-AGCTATGGTACTCCAGAAG ACCAGAGG-3′; IL-1b forward primer: 5′-GCAACTGTTCCTGAACTCAACT-3′; reverse primer : 5′- ATCTTTTGGGGTCCGTCAACT-3′.

Nissl Staining

Nissl staining was performed strictly according to the manufacturer's protocol (Solarbio, China). Briefly, brain slices with first incubated with Nissl Staining Solution for 30 min at 55 ℃, and then washed with 90 % ethanol and 50 % ethanol in order. Nikon fluorescence microscope (Ni-U) was used for observation.

siRNA-mediated interference

The following siRNA sequences were used: mouse SFXN1 sense 1: 5'-GAACGAACAGCUAGAGAAUtt-3'; mouse SFXN1 anti-sense 1: 5'-AUUCU CUAGCUGUUCGUUCtt-3'. mouse SFXN1 sense 2: 5'-GAACGAACAGCUAGAG AAUtt-3'; mouse SFXN1 anti-sense 2: 5'- UUGUUAAAGUACACACGCCtt-3'. human SFXN1 sense 1: 5'-GCUGCUAAUUGCAUUAAUAtt-3'; human SFXN1 anti-sense 1: 5'-UAUUAAUGCAAUUAGCAGCtt-3'. human SFXN1 sense 2: 5'- GUUG CAAGCUAAGAUCCAAtt -3'; human SFXN1 anti-sense 2: 5'- UUGGAUCUUAGC UUGCAACtt -3'.

Lactate dehydrogenase (LDH) release

The concentration of LDH released into the cell culture medium was measured with a

LDH assay kit (Beyotime, China). Cell supernatants were harvested and absorbance data was detected at 490 nm using a 96-well plate reader (Molecular Devices, USA). LDH release (%) was presented as the ratio of experimental LDH release to control LDH release in accordance with the manufacturer's protocols.

Immunofluorescence staining

Cultured cells were gently rinsed three times with PBS and then fixed in 4% paraformaldehyde (Solarbio, China). 0.5 % Triton X-100 was used to penetrated cells, and 5 % BSA was used for blocking at room temperature (1 h). Cells were then incubated with primary antibodies (anti-MAP2, Iba1, GFAP, SFXN1, 1:1000 dilution) overnight at 4 °C. After three washes in PBS, cells were incubated for 1 h at room temperature with secondary antibodies (Donkey anti-rabbit IgG-AlexaFluor-647, Donkey anti-mouse IgG-AlexaFluor-488 or Donkey anti-goat IgG-AlexaFluor-488, 1:1000 dilution). After PBS washing, cells were stained with DAPI (Solarbio, China) and were visualized with Nikon C2 microscope.

Brain samples were fixed in 4% PFA in phosphate buffer overnight at room temperature (RT) and equilibrated in phosphate buffer containing 30% sucrose for 48 h at 4 ℃. After permeabilization with 0.3 % triton-X-100 for 30 min, the cryosections were blocked by PBS containing 10 % serum for 2 h and treated with the specific primary antibody 4 ℃ overnight. After three times washes with PBS, the brain slices were stained with corresponding secondary antibodies for 2 h. DAPI mounting medium (Solarbio, China) was added to stain the nuclei. The brain slices were examined with Nikon C2 confocal microscope.

Detection of mitochondrial respiratory parameters

Mitochondrial function was assessed using the Seahorse XF96e Extracellular Flux Analyzer (Agilent, Santa Clara, CA, USA). SH-SY5Y cells were transfected with either siNC or siSFXN1 for 48 h, followed by OGD treatment for 2 h and reperfusion for 12 h. Then cells were collected and seeded at a density of 40,000 cells per well in an XF assay microplate and cultured overnight under standard conditions.

Prior to the assay, the culture medium was replaced with Seahorse XF assay medium (DMEM-based, pH 7.4, Agilent, supplemented with 10 mM glucose, 1 mM pyruvate, and 2 mM L-glutamine). Cells were equilibrated for 1 h at 37°C in a CO_2-free incubator to allow medium pH stabilization.

Following cell preparation, the microplate was loaded into the Seahorse XF96 Analyzer for a 2-hour assay. The oxygen consumption rate (OCR) was first measured at baseline to establish the fundamental respiratory activity. Subsequently, sequential measurements of OCR were performed following the injection of Oligomycin (1µM, an ATP synthase inhibitor), Carbonyl cyanide 4-(trifluoromethoxy)phenylhydrazone (FCCP, 1 µM, a mitochondrial uncoupler), Rotenone (0.5 µM) and Antimycin A (0.5 µM, a complex I and III inhibitors of the electron transport chain)[9, 10].

For data analysis, basal respiration was determined as the baseline OCR prior to oligomycin injection, ATP production was calculated by subtracting the minimal respiration (post-oligomycin OCR) from the basal respiration. All measurements were normalized to the total cell count per well to ensure comparability across samples.

References

[1] Chen HZ, Guo S, Li ZZ, Lu Y, Jiang DS, Zhang R, et al. A critical role for interferon regulatory factor 9 in cerebral ischemic stroke. J Neurosci. 2014;34(36):11897-912.1

[2] Xia CF, Smith RS, Jr., Shen B, Yang ZR, Borlongan CV, Chao L, et al. Postischemic brain injury is exacerbated in mice lacking the kinin B2 receptor. Hypertension (Dallas, Tex : 1979). 2006;47(4):752-61.2

[3] Quaegebeur A, Segura I, Schmieder R, Verdegem D, Decimo I, Bifari F, et al. Deletion or Inhibition of the Oxygen Sensor PHD1 Protects against Ischemic Stroke via Reprogramming of Neuronal Metabolism. Cell Metab. 2016;23(2):280-91.3

[4] Sasaki T, Takemori H, Yagita Y, Terasaki Y, Uebi T, Horike N, et al. SIK2 is a key regulator for neuronal survival after ischemia via TORC1-CREB. Neuron. 2011;69(1):106-19.4

[5] Schildge S, Bohrer C, Beck K, Schachtrup C. Isolation and culture of mouse cortical astrocytes. Journal of visualized experiments : JoVE. 2013(71).5

[6] Zhou X, Lv M, Duan Z, Liu W, Yan F, Liu J, et al. CHTOP Promotes Microglia-Mediated Inflammation by Regulating Cell Metabolism and Inflammatory Gene Expression. J Immunol. 2023.6

[7] Mihailovich M, Bremang M, Spadotto V, Musiani D, Vitale E, Varano G, et al. miR-17-92 fine-tunes MYC expression and function to ensure optimal B cell lymphoma growth. Nat Commun. 2015;6:8725.7

[8] Cui Y, Zhang Z, Zhou X, Zhao Z, Zhao R, Xu X, et al. Microglia and macrophage exhibit attenuated inflammatory response and ferroptosis resistance after RSL3 stimulation via increasing Nrf2 expression. J Neuroinflammation. 2021;18(1):249.8

[9] Damri O, Natour S, Agam G. Do Autophagy Enhancers/ROS Scavengers Alleviate Consequences of Mild Mitochondrial Dysfunction Induced in Neuronal-Derived Cells? Int J Mol Sci. 2021;22(11).9

[10] Manganelli V, Salvatori I, Costanzo M, Capozzi A, Caissutti D, Caterino M, et al. Overexpression of Neuroglobin Promotes Energy Metabolism and Autophagy Induction in Human Neuroblastoma SH-SY5Y Cells. Cells. 2021;10(12).10

Supplementary Figure legends

Figure S1. The expression of SFXN1 in the contralateral brain region after I/R injury.

(A, B) Representative immunoblot and quantification of SFXN1 in the contralateral region of the mouse brains after tMCAO followed by various time points of reperfusion, n=6. The data are means ± S.D., for all panels: n.s., no significance by One-way ANOVA analysis (B) followed by Tukey test.

Figure S2. The expression of SFXN1 in the primary cultured cells after OGD/R treatment

(A) Representative immunofluorescence staining of IBA1, SFXN1 and DAPI in primary microglia after OGD for 1.5 h and reoxygenation for indicated times, and analysis of SFXN1 intensity using relative fluorescence intensity per $IBA1^+$ cell. Scale bar, 10 μm. (B) Representative immunofluorescence staining of GFAP, SFXN1 and DAPI in primary astrocytes after OGD for 1.5 h and reoxygenation for indicated times, and analysis of SFXN1 intensity using relative fluorescence intensity per $GFAP^+$ cell. Scale bar, 10 μm. (C) Representative image showing immunofluorescence staining of MAP2, SFXN1, MitoTracker and DAPI in the primary neurons. Scale bar, 20 μm. The data are means ± S.D., for all panels: n.s., no significance, *P < 0.05, ***P < 0.001 by One-way ANOVA analysis (A-B) followed by Tukey test. All data are representative of or combined from at least three independent experiments.

Figure S3. Effects of SFXN1 siRNA-2 on cell viability of SH-5Y5Y cells after OGD/R.

(A) Representative immunoblot of SFXN1-2 in SH-SY5Y cells transfected with siNC or siSFXN1-2 for 48 h. (B) Cell viability (n=7) and (C) LDH release (n=6) was assessed and quantified in SH-SY5Y cells transfected with siNC or siSFXN1-2 for 48h followed by OGD treatment for 4 h and reperfusion for 24 h. The data are means ± S.D., for all panels: n.s., no significance, *P < 0.05, **P <0.01, ***P < 0.001 by Student's t-test (A) and Two-Way ANOVA analysis (B, C) followed by Sidak's multiple comparisons test. All data are representative of or combined from at least three independent experiments.

Figure S4. SFXN1 aggregate neuronal death after OGD/R in an iron-independent manner.

(A) Cell viability was assessed and quantified in SH-SY5Y cells transfected with siNC or siSFXN1 and subjected to OGD/R treatment in the presence or absence of DFO treatment for 24 h, n=8. (B) Flow cytometry analysis and (C) quantification of BODIPY in SH-SY5Y cells transfected with siNC or siSFXN1 for 48 h and subjected to OGD treatment for 3 h and reperfusion for 12 h, n=6. The data are means ± S.D., for all panels: n.s., no significance by Student's t-test (C) and Two-Way ANOVA analysis (A) followed by Sidak's multiple comparisons test.

Figure S5. Effects of SFXN1 siRNAs on inflammatory cytokine expression in

primary astrocytes and BV2 cells.

(A) Representative immunoblot of SFXN1 in primary astrocytes transfected with siNC or siSFXN1 for 48 h. (B) The qRT-PCR analysis of cytokine (TNFA, IL-1band IL-6) mRNA levels in siNC or siSFXN1-transfected primary astrocytes after OGD treatment for 3.5 h and reperfusion for 12 h, n=4. (C) Representative immunoblot of SFXN1 in BV2 microglial cells transfected with siNC or siSFXN1-2 for 48 h. (D) The qRT-PCR analysis of SFXN1 in BV2 microglial cells transfected with siNC or siSFXN1-2 for 48 h followed by LPS stimulation for 4 h, n=5. (E) The mRNA level of TNF-α, IL-6, and IL-1β in BV2 microglia-transfected with siNC or siSFXN1 followed by treatment with LPS for 4 h, n=4. The data are means ± S.D., for all panels: n.s., no significance, $*P < 0.05$, $**P < 0.01$ by Two-Way ANOVA analysis (B, D, E) followed by Bonferroni Test (B) or Sidak's multiple comparisons test (D-E). All data are representative of or combined from at least three independent experiments.

Figure S6. DFO rescues the reduced expression of proinflammatory cytokines and P-p65 after knocking down SFXN1.

(A, B) Flow cytometry analysis and quantification of DCFH-DA in BV2 cells transfected with siNC or siSFXN1 for LPS or PBS treatment, n=3. (C) The mRNA level of TNF-α, IL-6, and IL-1β in siNC or siSFXN1-transfected BV2 microglial cells followed by pretreatment with DFO 200 nM for 2 h and LPS stimulation for 4 h, n=6. (D) Representative immunoblot and statistical analysis of BV2 microglia transfected with siNC or siSFXN1 and 200 nM DFO treatment, after exposure to LPS for 0.5 h, n=3. Kruskal-Wallis test was used for (A). One-Way ANOVA analysis (C) was used followed by Bonferroni Test. All data are representative of or combined from at least three independent experiments.

Figure S7. Schematic diagram of lentivirus stereotaxic injection, and efficiency of AAV infection with brain cells.

Representative confocal image of colocalization analysis of viral EGFP with NeuN, Iba1 or GFAP and quantification of colocalization calculated as Pearson’s correlation coefficient, r. Scale bar, 100 µm. n=5. The data are means ± S.D., for all panels: $***P < 0.001$ by One-Way ANOVA analysis followed by Tukey test.

Supplementary Figure 1

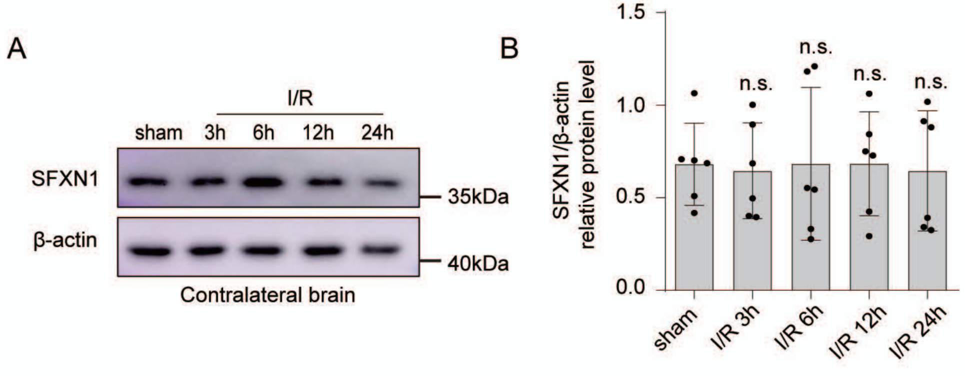

Supplementary Figure 2

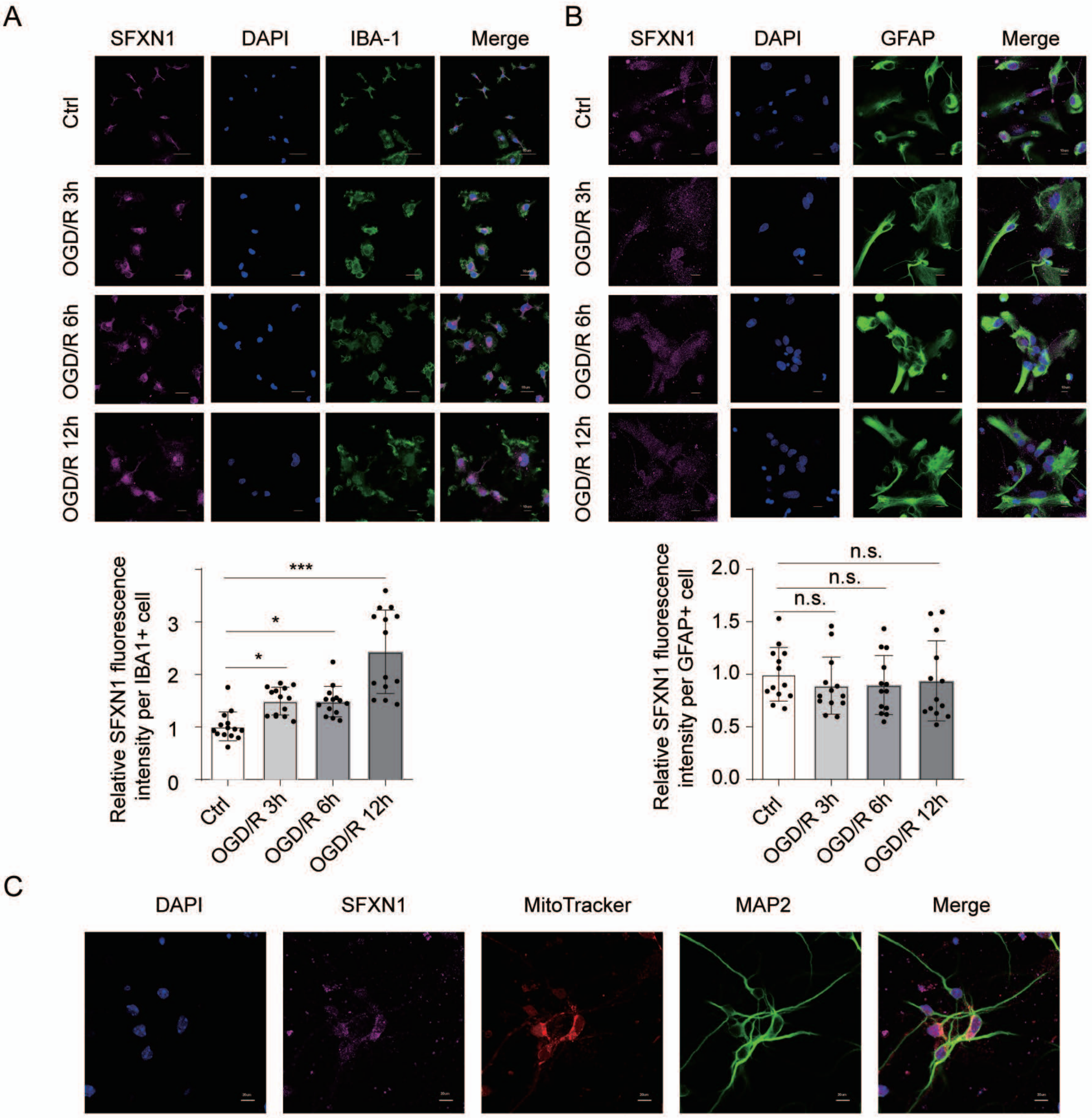

Supplementary Figure 3

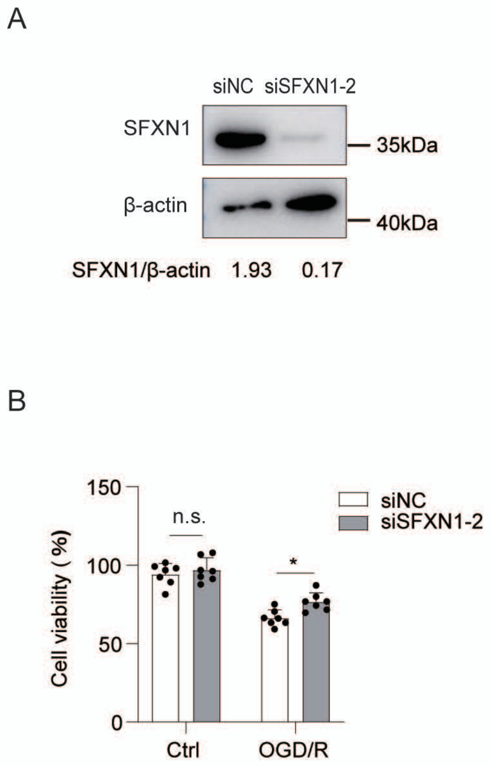

Supplementary Figure 4

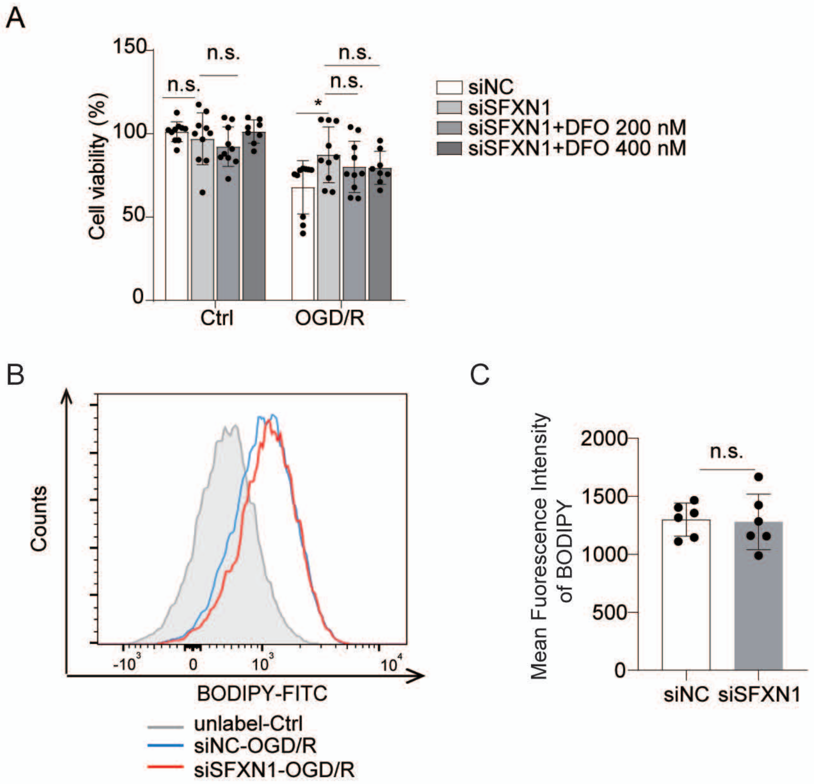

Supplementary Figure 5

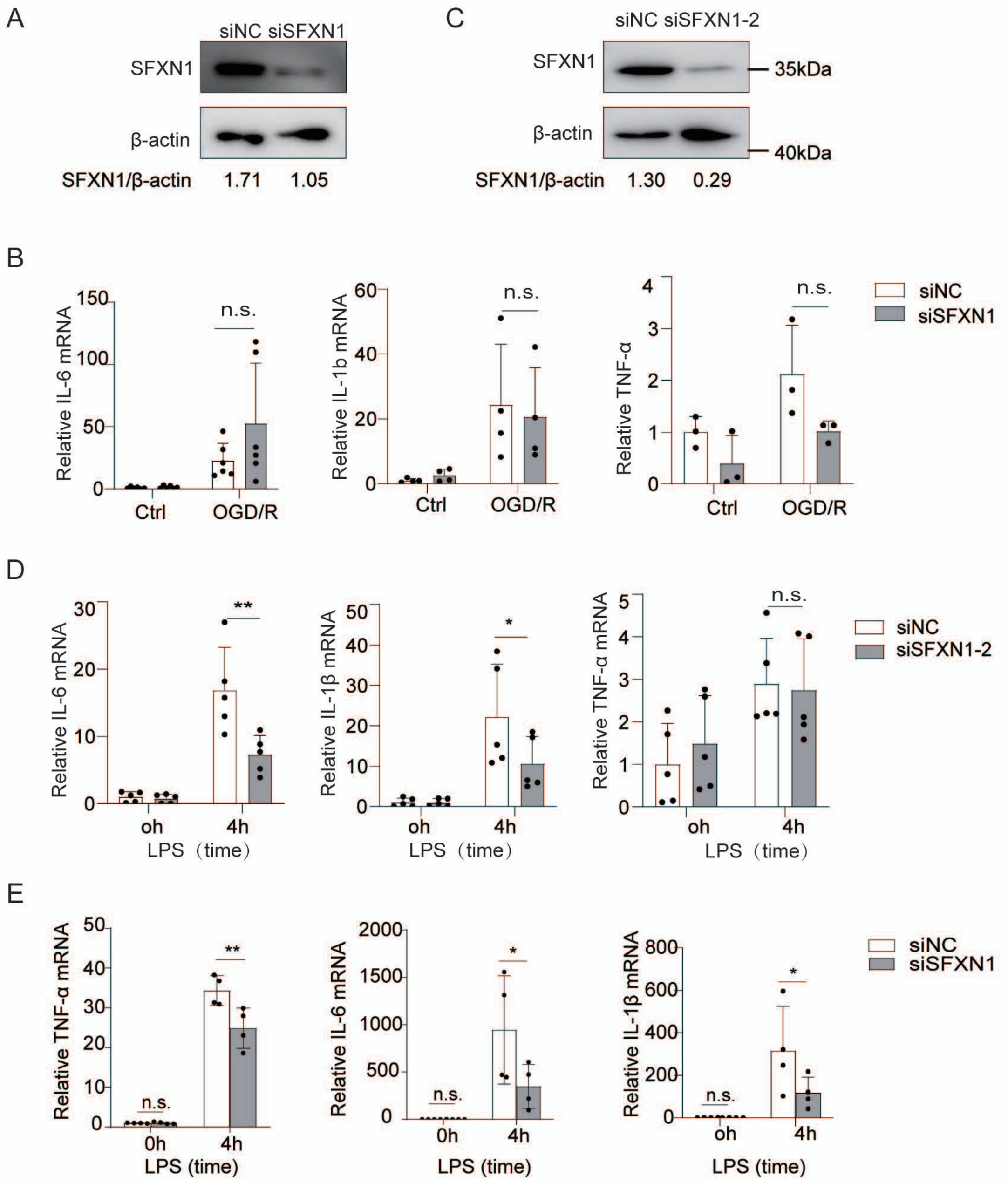

Supplementary Figure 6

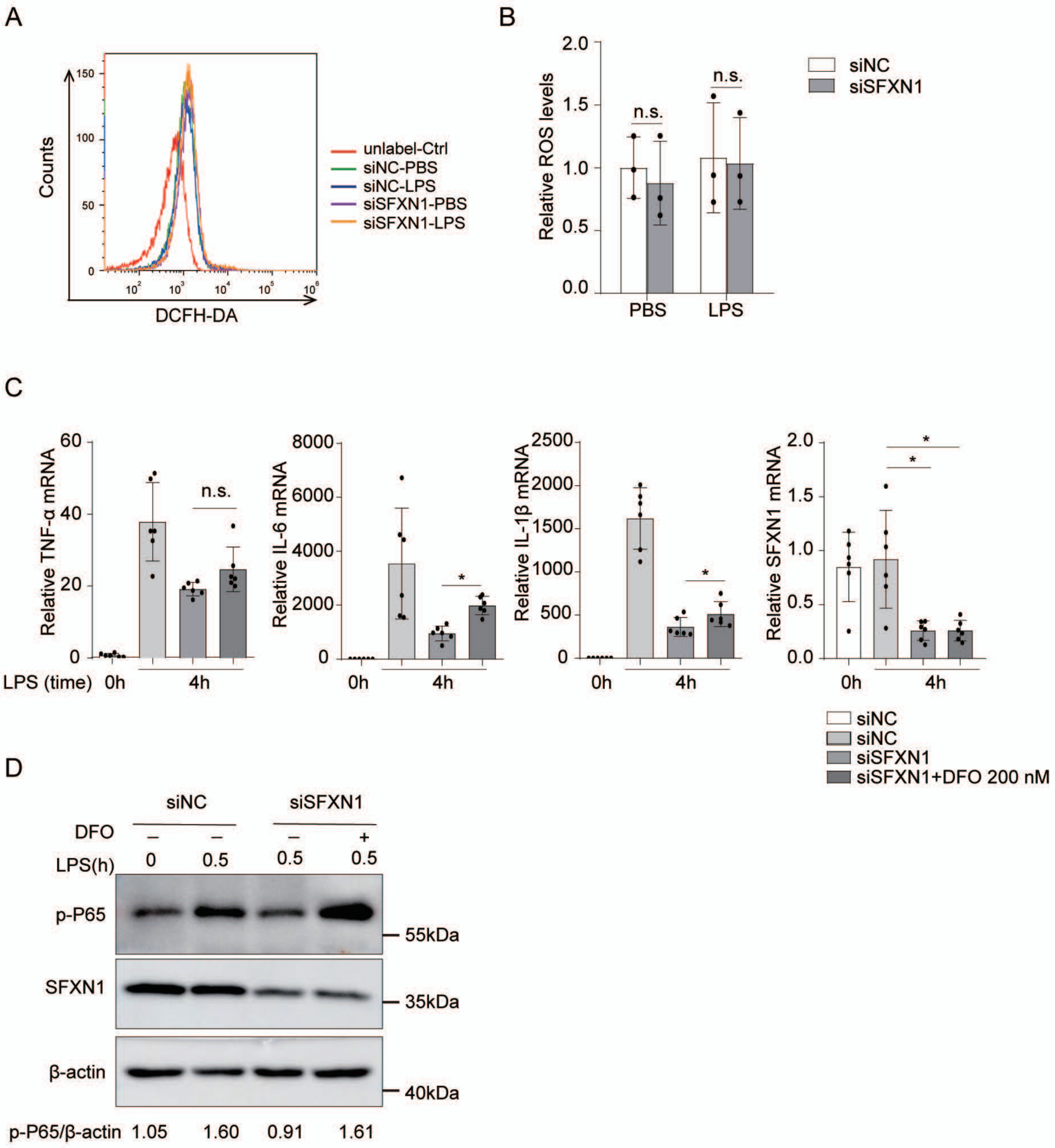

Supplementary Figure 7

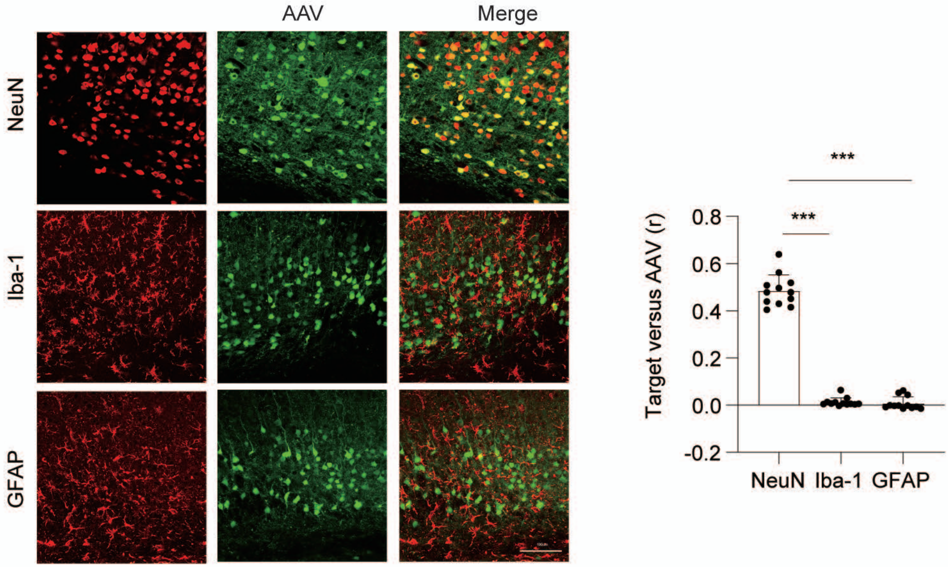

Supplement: Supplementary file 1 — Data S1. [file CNS-31-e70457-s001.pdf]
